# Soil multifunctionality and drought resistance are determined by plant structural traits in restoring grassland

**DOI:** 10.1002/ecy.2437

**Published:** 2018-08-20

**Authors:** Ellen L. Fry, Joanna Savage, Amy L. Hall, Simon Oakley, W. J. Pritchard, Nicholas J. Ostle, Richard F. Pywell, James M. Bullock, Richard D. Bardgett

**Affiliations:** ^1^ School of Earth and Environmental Sciences The University of Manchester Oxford Road Manchester M13 9PT United Kingdom; ^2^ NERC Centre for Ecology & Hydrology Wallingford OX10 8BB United Kingdom; ^3^ NERC Centre for Ecology & Hydrology Lancaster Environment Centre Lancaster LA1 4AP United Kingdom; ^4^ Lancaster Environment Centre Lancaster University Lancaster LA1 4YW United Kingdom

**Keywords:** aboveground–belowground interactions, carbon cycling, functional traits, plant–soil (belowground) interactions, resilience, restoration, root traits, soil microorganisms

## Abstract

It is increasingly recognized that belowground responses to vegetation change are closely linked to plant functional traits. However, our understanding is limited concerning the relative importance of different plant traits for soil functions and of the mechanisms by which traits influence soil properties in the real world. Here we test the hypothesis that taller species, or those with complex rooting structures, are associated with high rates of nutrient and carbon (C) cycling in grassland. We further hypothesized that communities dominated by species with deeper roots may be more resilient to drought. These hypotheses were tested in a 3‐yr grassland restoration experiment on degraded ex‐arable land in southern England. We sowed three trait‐based plant functional groups, assembled using database derived values of plant traits, and their combinations into bare soil. This formed a range of plant trait syndromes onto which we superimposed a simulated drought 2 yr after initial establishment. We found strong evidence that community weighted mean (CWM) of plant height is negatively associated with soil nitrogen cycling and availability and soil multifunctionality. We propose that this was due to an exploitative resource capture strategy that was inappropriate in shallow chalk soils. Further, complexity of root architecture was positively related to soil multifunctionality throughout the season, with fine fibrous roots being associated with greater rates of nutrient cycling. Drought resistance of soil functions including ecosystem respiration, mineralization, and nitrification were positively related to functional divergence of rooting depth, indicating that, in shallow chalk soils, a range of water capture strategies is necessary to maintain functions. Finally, after 3 yr of the experiment, we did not detect any links between the plant traits and microbial communities, supporting the finding that traits based on plant structure and resource foraging capacity are the main variables driving soil function in the early years of grassland conversion. We suggest that screening recently restored grassland communities for potential soil multifunctionality and drought resilience may be possible based on rooting architecture and plant height. These results indicate that informed assembly of plant communities based on plant traits could aid in the restoration of functioning in degraded soil.

## Introduction

Aboveground plant functional traits have been shown to predict a wide range of soil processes and services, including nutrient cycling (de Vries and Bardgett 2016), carbon (C) storage (Fornara and Tilman 2008) and resilience of ecosystem functions to drought (Isbell et al. 2015, 2017). Broadly, traits of aboveground organs have been found to fit along a spectrum of resource economics, from fast‐growing, exploitative species, to slow‐growing, conservative species (Wright et al. 2004). It has been proposed that belowground responses are more exactly explained by root traits, which form a direct interface between plants and soil (Bardgett et al. 2014, Gould et al. 2016, Legay et al. 2016). However, there is an increasing body of evidence that shows that roots may not adhere to the same resource economics spectrum as leaves and shoots, or at least, a root economics spectrum would be unlikely to align with that of leaves (Erktan et al. 2018). Roots have different resource acquisition strategies, microbial associations and spatial and temporal turnover to leaves, and at least two axes of variation in root traits have been identified (Kong et al. 2014, Weemstra et al. 2016, Erktan et al. 2018). When attempting to characterize or predict soil functions using plant functional traits, it is crucial that both above and belowground plant traits are included.

Despite growing interest in the potential for plant traits to influence soil functions, many gaps in our understanding remain. One gap is our knowledge of the resilience of soil functions to environmental perturbations, such as climate extremes under field conditions (Bardgett 2017, Laliberté 2017). Another important gap is the applicability of traits derived from databases in real‐world scenarios. Trait databases are potentially a hugely important resource, but root traits are still under‐represented, and the traits that are included have not been tested for predictive power of soil functions in large‐scale experimental studies. Indeed, traits such as root architecture, mycorrhizal affinity, and depth class have the potential to be used in simple mechanistic hypotheses that describe their role in soil functioning without the need for in situ measurements, when included alongside aboveground traits (Kleyer et al. 2008, Messier et al. 2017). Finally, as the climate changes, relationships between plant traits and soil functions are likely to change (Fry et al. 2013, Sayer et al. 2017). While evidence is building for the decoupling of trait–function relationships under drought, as before, root traits are overlooked. The incomplete understanding of the role of plant traits, and especially root traits, as regulators of soil functions and their response to perturbations in the real world represents an important hurdle to challenges concerning sustainable soil management and restoration of degraded soils (Bardgett 2017).

Here, our goal was to test the hypothesis that variation in soil functions related to C and nitrogen (N) cycling, and their resilience to drought, are closely linked to the presence of particular trait‐based functional groups. Further, we tested whether trait values from databases, particularly root traits, are useful in predicting soil functioning, multifunctionality and resistance of soil functions to drought. We hypothesized that more complex root architectures are more likely to be associated with higher rates of nutrient cycling and nutrient availability due to increased microbial niche heterogeneity and better foraging ability than simpler tap rooted species. We also hypothesized that deeper roots may increase resistance to drought be accessing water in deeper soil layers. To address our hypotheses, we set up a 3‐yr field experiment at Salisbury Plain, southern England, where three plant species groups assembled according to database values for key traits, including rooting depth class and root architectural class, were sown into degraded ex‐arable land to form a gradient of plant functional and species diversity. Trait‐based groups were selected from plant assemblages typical of species‐rich chalk grasslands on the basis of their expected effects on soil functions. By taking this approach, our study advances on past research that has examined relationships between plant functional traits and soil functions, in that we explicitly test how plant communities assembled on the basis of different trait‐based functional groups, and their combinations, impact soil functions and multifunctionality, and their resilience to climate extremes under the real world scenario of grassland restoration. Further, we focus on the initial stages of restoration when the rapid recovery of soil functionality and resilience is likely to influence longer‐term restoration success (Fry et al. 2017b).

## Methods

### Experimental design

The field experiment was established in 2013 at Winklebury Hill, Wiltshire, southern England (50.5988° N, 2.0709° E, 260 m above sea level), on an area of calcareous land that had been under continuous cultivation (spring barley and winter wheat rotation) since 1995 (see Appendix S1 for detailed methods). The experiment used a randomized factorial block design with plots containing plant species groups based on functional effects traits; these encompassed a range of contrasting trait groups, and were constructed such that plant species and functional diversity were both varied. We designed the trait group classification following the technique outlined by Fry et al. (2014), using above‐ and belowground plant trait data from the Leda traitbase (Kleyer et al. 2008), Ecoflora database (Fitter and Peat 1994), Grime et al. (2007), and PlantATT (Hill et al. 2004). Trait data were selected to separate species into groups with hypothetically contrasting impacts on soil C and N functioning (Table 1).

**Table 1:** Summary table showing the database traits used in the classification and the rationale for their use

Trait	Data format	Link with function	Supporting references
Plant height^a^	continuous	Proxy for shading and aboveground competition. Closely linked with photosynthetic rate. Positively related to a more acquisitive resource use strategy.	Ostertag et al. (2015); De la Riva et al. (2016)
Perenniality^a^	ordinal scale from annual to perennial (5 levels)	Describes both C residence time and intervals between large inputs. Potentially describes the speed of nutrient cycling and decomposition.	Knops et al. (2002)
Specific leaf area (SLA)^b^	continuous	Key trait in leaf economics spectrum. Positively related to a more acquisitive resource use strategy.	Wright et al. (2004); De Vries et al. (2012); Manning et al. (2015)
Root architecture^a^	categorical from fibrous (complex) to tap rooted (simple) (eight levels)	A gradient from highly complex, acquisitive roots that collect more water and nutrients from the soil, to simple roots that act as C storage structures and resist drought. Complex roots may improve soil aggregation.	Aerts et al. (1992); Buckland et al. (1997); Gould et al. (2016); Messier et al. (2017)
Rooting depth^c^	ordinal scale from shallow to deep (three levels)	Indicates whether root exudates and sloughing will occur mainly in the warm shallow soil layers or the cooler, more anoxic deeper layers where microbial activity is low. Plant derived C for decomposition may be unavailable if placed in deep layers. Deep roots often confer drought tolerance.	Morecroft et al. (2004); Fay et al. (2008)
Arbuscular mycorrhizal (AM) affinity^c^	ordinal scale, from usually mycorrhizal, sometimes mycorrhizal, to never mycorrhizal (three levels)	Implications for nutrient cycling and soil C inputs.	Marschner and Dell (1994)

*Notes*

a PlantATT (Hill et al. 2004).

b Leda traitbase (Kleyer et al. 2008).

c Ecoflora (Fitter and Peat 1994).

The species used were selected from the calcareous grassland CG3a plant community type (*Bromus erectus* grassland with typical sub‐community, UK National Vegetation Classification; Rodwell 1992), which is the dominant grassland community of the region and is typically used as a target community in restoration programs (Pywell et al. 2002). Plant species were classified into three groups by divisive hierarchical cluster analysis using the cluster package (hereafter FG1, FG2, and FG3; Maechler et al. 2015) in R 3.1.0 (R Core Team 2015). These three groups determined dissimilarities in the trait data using Ward's distance measure (see Fry et al. 2014 for further details). The classification of each species into the three groups was then verified using linear discriminant analysis (LDA) to ensure that all species were correctly assigned.

The experimental design formed a gradient of all combinations of the three functional groups with each group alone, all paired combinations, and all three groups in one plot. This resulted in seven treatments, each replicated six times, once in each block in a randomly allocated position (*n* = 42). Plots were 8 × 8 m and separated by 2 m on each side. Seeds and plug plants were sown into the plots in May 2013. See Appendix S1: Table S1 for full species lists.

### Drought treatments

In 2015, we set up rainout shelters (1 × 1.5 m) to impose drought on each plot. For this, two shelters were erected per plot: one for rainfall exclusion, while the other had ~30 holes of 5 cm diameter to account for unintended microclimate effects while allowing most rainfall through. This was used to account for roof‐induced artifacts on humidity, light, and temperature (Vogel et al. 2012). A matching control area in each plot had no roof. Therefore, there were three subplots per plot, and all measures were taken from each subplot (126 subplots). The drought regime was designed by taking 10‐yr daily rainfall data from Yeovilton air base (29 miles away) in May–August between 2004–2014, and using a Gumbel distribution to determine a severe drought event that would occur once in 100 yr (VGAM package [Yee 2010]; rainfall data *available online*).^6^ This was calculated to extend over 41 d, so the shelters were in place between 1 June and 13 July 2015. During the drought period, soil moisture reached permanent wilt point under full shelters, with an average of 5.01% soil moisture content.

### Vegetation characteristics

In May and July of 2015 (before and after the shelters were in place), we conducted a vegetation survey using a permanent 50 × 50 cm quadrat in each subplot and a visual estimation of percentage cover of litter (unattached dead material), bryophytes, and individual plant species. We calculated functional diversity indices using database values (community weighted mean [CWM] and functional divergence [FDvar] of each trait; see Table 1 for trait details) and biomass assessments (Appendix S1 for more details).

### Soil properties

Soil samples were taken in each subplot the week before the shelters were erected (May), and immediately after the shelters were removed (mid‐July) to capture the response of communities and functions to the drought. Samples were also collected eight weeks after shelter removal (mid‐September), to determine recovery before the vegetation senesced. Soil samples were analyzed for a suite of properties, which were all expressed on a unit area basis based on bulk density assessment. Plant‐available ammonium (NH_4_‐N), dissolved organic C (DOC) and inorganic N (NO_3_‐N), Olsen's extractable P (PO_4_‐P), and mineralization and nitrification rates were measured using standard methodology. Microbial C and N were measured using the chloroform‐fumigation technique, and microbial biomass was measured using phospholipid fatty acid (PLFA) analyses. Decomposition rates were measured using litter bags. These were stapled to the soil surface in late May 2015, two per subplot, and one was removed in mid‐July 2015 and the other in September 2015 (see Appendix S1 for detailed methods). Raw data may be found in Fry et al. (2017a).

### CO_2_ fluxes

Each month from May to September 2015, CO_2_ flux measures were taken from each subplot using an infra‐red gas analyzer (IRGA; EGM4, PP Systems, Hitchin, UK). Circular gas sampling collars (25 cm diameter) were inserted into the soil surface in April. At each measurement time, clear flux chambers were sealed over the collars (total volume 10,454 cm^3^; Ward et al. 2007). Measures were taken in the light for two minutes to measure Net Ecosystem CO_2_ Exchange (NEE), then in the dark using a reflective cover for a further two minutes to measure Ecosystem respiration (*R*_eco_). *R*_eco_ was subtracted from NEE to gain an estimate of photosynthetic rate (*P*_syn_). Concurrent volumetric soil moisture content (SMC), soil temperature and mean photosynthetically active radiation (PAR) measures were taken in triplicate (temperature and moisture measured using a WET sensor, Delta‐T Instruments, Cambridge, UK; PAR measured using a light meter, Skye Instruments, Llandridod Wells, UK).

### Statistical analysis

All analyses were carried out using R3.4.1 (R Core Team, 2015). First, we validated the functional group classification in each plot. Differences between mean database‐derived trait values in each plant functional group were assessed using one‐way ANOVA, with the trait values for each plant species as the response variable and the group identifier (one, two, or three as factors) as the explanatory variable. To assess whether distinct communities were achieved in the field, divergence in plant group composition was assessed by using percentage cover values for each species in a nonmetric multidimensional scaling (NMDS) ordination using Bray‐Curtis distance measures and 1,000 permutations (Kruskal 1964). Bray‐Curtis was chosen because it can account for rare taxa with many zero values (Zibulski et al. 2016). We then used envfit (NMDS and envfit were from the vegan package; Oksanen et al. 2010) to evaluate whether the plant functional group treatment had resulted in discrete species assemblages. We assessed whether there was a difference in trait distributions in the plots by using the CWM and FDvar values as response variables in linear mixed effects models (lme), with roof and functional diversity treatment as explanatory factors and block and plot as random effects. FDvar traits were logit transformed.

We then carried out analyses to test whether the treatments affected the soil function measures over the course of the summer season using repeated‐measures ANOVA, with each function tested in turn; month, roof, and functional group treatment and their interactions as the fixed effects; and block and plot as random effects. In order to analyze the effects of realized functional traits on these functions, we followed the protocol described by Feld et al. (2016), first using Random Forest analysis to find the most appropriate traits for each function, using the randomForestSRC package in R (Ishwaran and Kogalur 2018). We included CWM and FDvar of all six traits chosen for the initial grouping. We also included soil moisture and the roof treatment in each Random Forest model. All data were scaled and transformed where necessary, and outliers subjected to the recommended removal protocol by Feld et al. Random Forest does not work for repeated measures, so we carried out the analysis separately for each function and each time point (May, July, and September 2015). We chose the traits with the highest percent increase of mean squared error (MSE) for downstream analysis, and then used month, the chosen traits, and any interactions that had been identified in a repeated measures lme as fixed effects. Using the dredge function in the MuMin package (Bartón 2018), we used multimodel inference to calculate all models possible with the given fixed effects, and selected those with the threshold of ΔAIC< 2 from the best model. Where more than one model was selected, we used model averaging to select the best model subset. All model validation was carried out as recommended by Feld et al. (2016).

Next we tested whether the functional traits described multifunctionality of the soil system. We used the method presented by Manning et al. (2018) to calculate thresholds of soil multifunctionality for each function measured. We calculated these separately for May, July, (after the shelters were removed) and September (eight weeks after the shelters were removed), because the values for function were likely to change through the season. Briefly, the analysis uses a cluster analysis to group the functions, then weightings are applied to each function so all functions in a given cluster sum to one. A new data set is created where the values for each function are substituted for either the weighting value, if the function crosses the threshold of being in the top 50% for that plot, or zero. All values are summed so each subplot has a single multifunctionality value, with a potential maximum of one. We then ran linear mixed effects models (lmes) to test which traits were the most important for determining multifunctionality at each time point, and employed the same model structure and multimodel inference as before.

Next we assessed the impact of the rain shelters on soil functions. Resilience comprises resistance to and recovery from a perturbation (Oliver et al. 2015); these were calculated using the metrics presented by Orwin and Wardle (2004). We calculated resistance in terms of the roofed drought treatment compared to the unroofed control in July when they were removed. The equation used was as follows: RS(*t*_0_) = 1 − 2|*D*_0_|(*C*_0_ + |*D*_0_|), where *C*_0_ is the value of the soil function of the unroofed control in July when the roofs were removed, while *D*_0_ is the value of the roofed drought treatment in July. We calculated recovery by comparing values in September with those obtained shortly after the roofs were removed in July. The resilience metric uses the same values as above for *D*_0_, while *D*_*x*_ is the difference between the unroofed control in September after the recovery period, and the roofed drought treatment. The equation is thus RL(*t*_*x*_) = 2|*D*_0_|(|*D*_0_|+|*D*_*x*_|)−1. Both metrics are bounded between −1 and 1. A value of 1 indicates complete resilience, 0 means no resilience, and negative values mean drought values are higher than the control. Finally, we assessed resilience of soil functions to drought using one‐way ANOVA to assess whether the values were different to 1 or zero, followed by the lme model structure as above to assess plant attribute effects. The response variables used were belowground biomass, CO_2_ fluxes, decomposition, rates of N mineralization and nitrification, microbial biomass C and N, and soil DOC, inorganic N, and P.

## Results

### Functional group validation

The cluster analysis identified three plant functional groups with contrasting trait syndromes, thereby enabling us to test our hypotheses about the individual and combined effects of trait‐based functional groups on ecosystem and soil functions (see Appendix S1: Table S1 for full species lists). The first group (hereafter FG1) contained 34 species, of which 21 were available to be planted in the site. FG1 species had a much wider range of lifespans than the other two groups, which tended to be mostly perennials (Appendix S1: Fig. S1; *F*_2,80 _= 7.25, *P* = 0.001). FG1 also had deeper roots (*F*_2,80 _= 47.22, *P* < 0.001), a wider range of rooting architectures than the other two, and on average were simple tap‐rooted structures (*F*_2,80 _= 47.03, *P* < 0.001; Fig. S1). We therefore inferred that this group would have lower C and N cycling rates as there were fewer fine absorptive roots and fewer niches for microbes to associate with, but that they may be more resilient to drought as they could reach deep into the chalk. Group two (FG2) contained 29 species, of which 16 were planted, and was characterized by plant species that were significantly smaller in height than the other two (*F*_2,80 _= 6.30, *P* = 0.003). These species were long lived and had shallow simple tap roots (*F*_2,80 _= 47.03, *P* < 0.001). Many of these were rosette forbs. We suggest that these would have few impacts on nutrient cycling as their roots have a very small sphere of influence. Finally, group three (FG3) contained 33 species and 22 were planted; it had shallow complex root architectures (Appendix S1: Fig. S1; *F*_2,80 _= 47.03, *P* < 0.001) and a lower specific leaf area than the other groups (*F*_2,80 _= 7.50, *P* = 0.001), indicating thick fleshy leaves. These are likely to have many niches for microbial association and thus high levels of nutrient and C cycling. Verification of these groups by linear discriminant analysis showed agreement of 93.8%. All groups contained a mixture of grasses, forbs, and legumes.

### Vegetation characteristics

**Figure 1:**
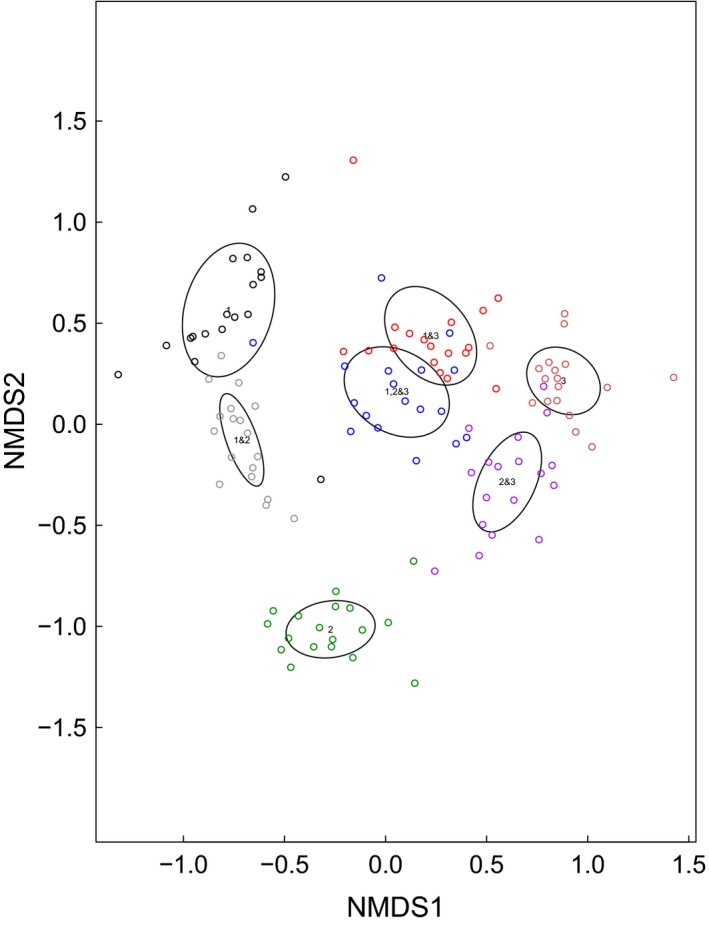
Nonmetric multidimensional scaling (NMDS) ordination of species composition in July 2015. Ellipses are standard deviation from the mean. Each ellipse and color represents a different functional group combination, and each dot represents a subplot.

**Figure 2:**
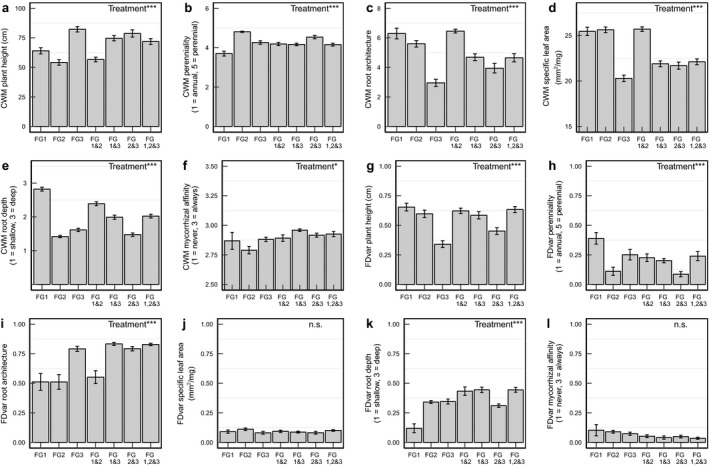
(a–f) Realized community weighted trait means (CWM) and (g–l) functional divergence (FDvar) of each functional group treatment in July 2015. Treatments are seven functional group combinations. Error bars are SEM. *P < 0.05; **P < 0.01; ***P < 0.001; n.s., not significant.

By 2015, NMDS ordination showed that the seeding treatments had created seven distinct plant communities (Fig. 1), with discrete trait‐based groups. In total, 116 species grew in the plots in 2015, with an average of 11 species per plot. CWM traits were unique to each group or combination of groups, so contrasting trait syndromes were achieved across treatment plots (Fig. 2). Aboveground biomass was significantly higher when FG3 was present in the plots in September (Appendix S1: Fig. S4; 630.86 g/m^2^ when present compared with 452.74 g/m^2^), but there was no effect of the roof treatment (*F*_1,30 _= 11.64, *P* = 0.002). Belowground biomass, measured using ingrowth cores, showed a significant interaction between FG3 and the roof treatment in both July and September. Under control conditions, all vegetation treatments had similar root biomass (122.90 g/m^2^ in July, 210.65 g/m^2^ in September) but, in roofed subplots, root biomass was significantly lower when FG3 was present compared with when it was absent (in July 57.64 g/m^2^ when present vs. 76.60 g/m^2^; in September 177.29 g/m^2^ when present vs. 207.05 g/m^2^; *F*_2,112 _= 5.98, *P* = 0.003; Appendix S1: Fig. S4).

While there was no overall effect of the drought treatment on plant species composition, some individual species were suppressed, such as *Galium mollugo*, which was reduced under both roofed treatments, particularly when there was more than one functional group present (*F*_2,222 _= 4.72, *P* = 0.010). Cover of *Rumex acetosa* was also lower under the shelters where present (*F*_12,222 _= 2.61, *P* = 0.003), whereas *Bromus commutatus* and *Dactylis glomerata* were more abundant under shelters (*F*_12,222 _= 2.24, *P* = 0.011 and *F*_2,222 _= 3.72, *P* = 0.026, respectively).

### Plant trait effects on individual functions

**Figure 3:**
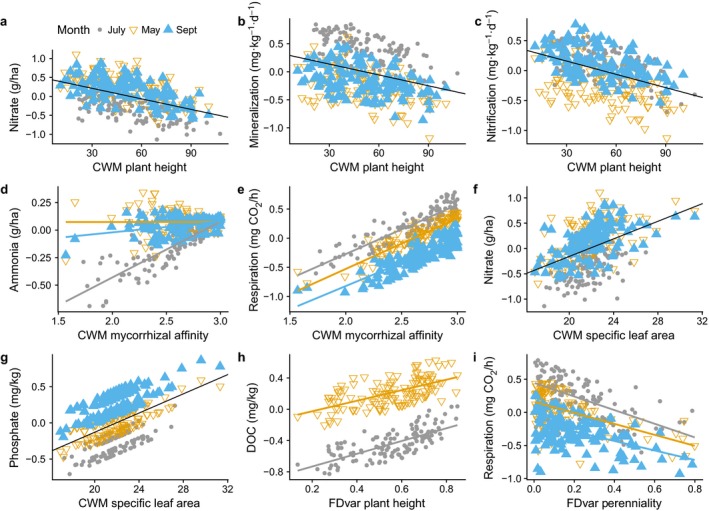
Repeated‐measures analyses of the effect of plant functional traits on soil functions in May, July, and September 2015. Soil functions have been standardized and data shown are predicted values derived from linear mixed effects models. When the effect of month is non‐significant, one line of best fit is used for all data. Mycorrhizal affinity values are bounded between 1 (never mycorrhizal) and 3 (always mycorrhizal). FDvar is a measure of functional divergence, and is bounded between 0 (no variation in the trait) and 1 (every value is different). DOC, dissolved organic carbon.

The relationships between individual soil functions and plant traits were most strongly explained by plant height, mycorrhizal affinity, and specific leaf area (Fig. 3; Appendix S1: Table S2). Inspection of the trait–function relationships at different time points using Random Forest analysis revealed that when plots were dominated by taller species, there were lower mineralization (Fig. 3b; *F*_1,370 _= 13.79, *P* < 0.001) and nitrification rates (Fig. 3c; *F*_1,369 _= 15.32, *P* < 0.001), leading to a decrease in soil NO_3_‐N availability (Fig. 3a; *F*_1,368 _= 23.32, *P* < 0.001). Inspection of the different time points measured revealed that this was chiefly observed in July and September, after biomass had peaked and begun to senesce. Increased CWM mycorrhizal affinity resulted in a positive effect on plant‐available NH_4_‐N (Fig. 3d; Month × SMC × CWM Mycorrhizal affinity, *F*_1,360 _= 17.27, *P* < 0.001), which was strongest in July, and increased *R*_eco_ (Fig. 3e; *F*_1,369 _= 26.79, *P* < 0.001). CWM specific leaf area was positively related to soil NO_3_‐N availability (Fig. 3f; *F*_1,368 _= 15.50, *P* < 0.001) and phosphate (Fig. 3g; *F*_1,370 _= 7.62, *P* = 0.006), so larger, less dense leaves were associated with higher soil nutrient availability. FDvar plant height was positively associated with soil dissolved organic C (DOC) concentration, indicating that there was more DOC available when there was a mixture of plant heights in the plot (Fig. 3h; *F*_1,243 _= 6.14, *P* = 0.014). *R*_eco_ was negatively associated with FDvar perenniality, so if a plot had a larger mix of longevities, *R*_eco_ was lower overall (Fig. 3i; *F*_1,369 _= 4.73, *P* = 0.030). Finally, there was no significant effect of plant traits on the microbial community, as measured by PLFA.

### Plant trait effects on multifunctionality

**Figure 4:**
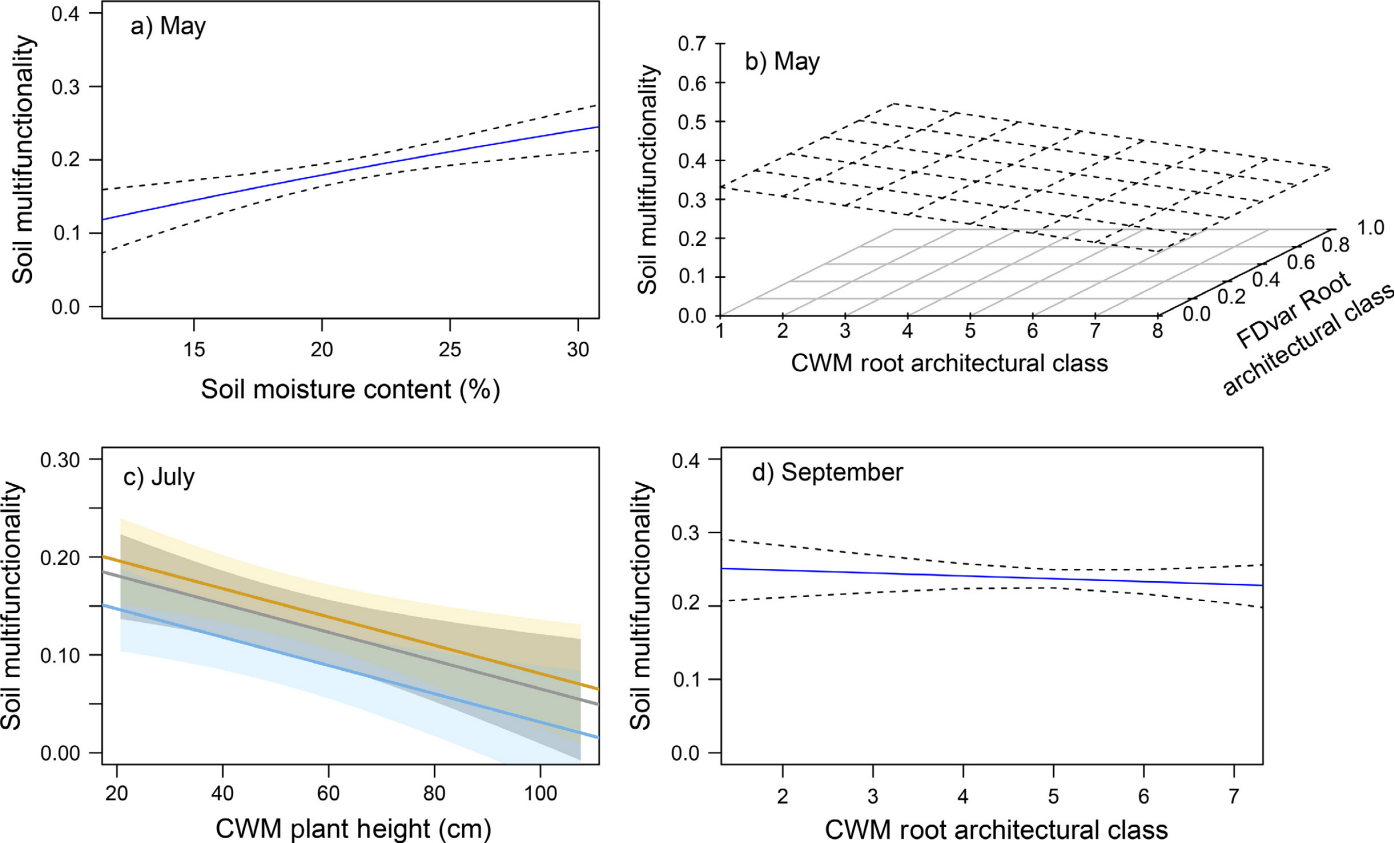
Effects of soil moisture and plant functional traits on soil multifunctionality, derived from threshold values of each function calculated using the metric presented by Manning et al. (2018). Graphs represent predicted values based on significant model fits in (a, b) May, (c) July, and (d) September 2015. Root architectural class is a categorical variable ranging from 1 (fine fibrous roots [very complex]) to 8 (tap roots [very simple]). In July, best fit lines represent differing effects of the roof treatment, where yellow is roofed control, gray is drought, and blue is unroofed control. The shaded polygons represent standard error of the mean.

Soil multifunctionality was associated with plant architectural traits through the season, namely plant height and root architectural class (Fig. 4). At the end of May, as the vegetation reached peak biomass, soil moisture was the most important descriptor of multifunctionality, with higher multifunctionality values in wetter plots (Fig. 4a; *F*_1,117 _= 13.66, *P* < 0.001). However, both the CWM and divergence (FDvar) of root architectural class were also important in May, with the highest values of multifunctionality occurring in plots with fine fibrous roots and high variation in root architecture across the plots (Fig. 4b; *F*_1,117 _= 6.90, *P* = 0.010). Conversely, plots uniformly dominated by simple tap‐rooted species had much lower values of multifunctionality. By July, average multifunctionality values were lower than in May or September (Fig. 4c). In July, CWM plant height and roofing treatment were the most significant descriptors of multifunctionality. Droughted and roofed control plots had higher values of multifunctionality than the unroofed control, and this declined to close to zero when plots were dominated by taller species (*F*_1,120 _= 8.49, *P* = 0.004). By September, multifunctionality values were very consistent across all plots. However, multifunctionality was lower in plots dominated by species with more complex, fibrous rooting architecture (lower CWM root architecture values) compared with plots dominated by species with simpler tap‐rooted architectures, similarly to values in May.

### Resilience

**Figure 5:**
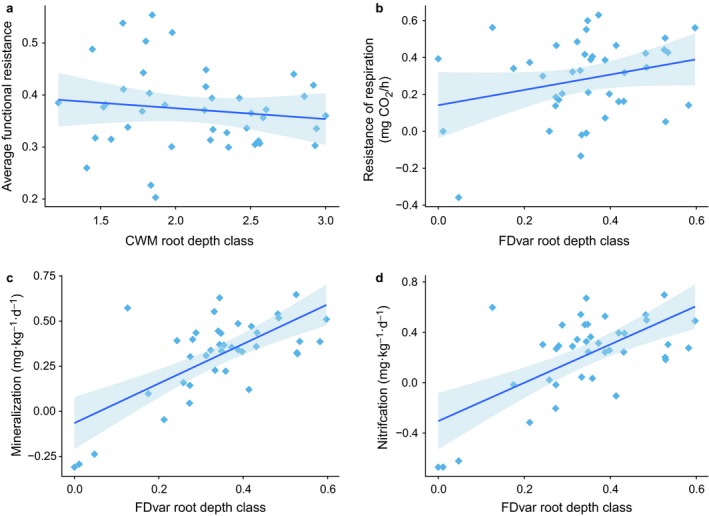
Effects of plant functional traits on resistance of soil functions to drought. Resistance is calculated using the metric of Orwin and Wardle (2004). A resistance value of 1 represents full resistance or no difference from the control when drought ends in July, 0 denotes a 100% change, while negative numbers indicate that the droughted value is higher than the control value. Root depth class is bounded between 1 (shallow) and 3 (deep).

At the end of the drought treatment, soil moisture was reduced by an average of 33.96% (26.08% SMC under unroofed control, compared with 17.22% SMC under full rain shelters). Resistance of soil functions, measured in July on the same day the shelters were removed, were consistently significantly different from 1 (complete resistance to drought) for every plant functional group treatment, so there was no complete resistance observed. When compared to 0 (100% change in the variable), both soil NH_4_‐N and NO_3_‐N were statistically similar to zero for all plant functional groups individually. Linear mixed effects models revealed that the average resistance of all functions was significantly related to the CWM of root depth class (shallow, medium, deep), where shallow roots were associated with higher resistance to drought (Fig. 5a; *F*_1,32 _= 6.07, *P* = 0.019). When individual soil functions were evaluated, variation in root depth (FDvar) was consistently the most important indicator of resistance. We observed that functions associated with nutrient turnover and cycling were more resistant to drought when there was a mixture of root depth classes in the plot (Fig. 5b–d). This was true of *R*_eco_ (*F*_1,32 _= 1,31, *P* = 0.009), and rates of N mineralization (*F*_1,31 _= 7.52, *P* = 0.010) and nitrification (*F*_1,31 _= 10.32, *P* = 0.003).

**Figure 6:**
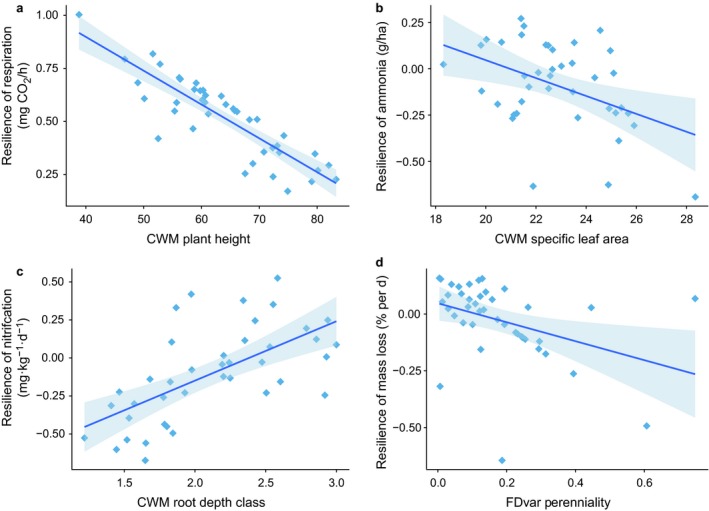
Effects of plant functional traits on resilience of soil functions to drought eight weeks after roofs were removed. Resilience is calculated using the metric of Orwin and Wardle (2004). A resilience value of 1 represents full resilience, or no difference from the control after eight weeks of recovery. 0 denotes a 100% change, while negative numbers indicate that the droughted value is higher than the control value.

Recovery of the system, defined as droughted soil function values relative to the level of the control, was not complete eight weeks after the drought. Most variables were not significantly different from zero regardless of plant functional group presence in the subplots. This includes belowground biomass, photosynthetic rate, decomposition rate, DOC, and rates of N mineralization and nitrification, soil NH_4_‐N and NO_3_‐N, and phosphate, so drought values were nearly as distant from the control as they had been at the end of the drought. When the values for resilience were averaged across all soil functions, there was no significant relationship with any plant trait. This occurred because resilience of each individual function was explained by a different trait. For example, resilience of *R*_eco_ was lower in plots dominated by tall plants (Fig. 6a; CWM plant height; *F*_1,34 _= 8.73, *P* = 0.006). Resilience of soil NH_4_‐N concentrations was lower when plots were dominated by species with high specific leaf area, so recovery was lower when leaves were large and thin (Fig. 6b; CWM SLA; *F*_1,32 _= 7.05, *P* = 0.012). However, nitrification rate was more resilient to drought in plots dominated with deeper rooted species (Fig. 6c; CWM root depth class; *F*_1,31 _= 14.04, *P* < 0.001). Finally, resilience of decomposition was highest in plots where all plants had approximately similar longevity, while increased variability in lifespan led to decomposition that was higher than control values (negative resilience values indicating an overshoot; Fig. 6d; *F*_1,34 _= 6.40, *P* = 0.016).

## Discussion

Here we present the findings of a large‐scale, field‐based experiment that demonstrates that plant height and structural root traits, i.e., architecture and depth class, act as important determinants of individual soil functions and multifunctionality during the early stages of grassland restoration. Further, we have identified key plant‐based drivers of soil functions throughout the growing season, and the role of root traits in determining the resistance and resilience of these soil functions to severe drought. Our approach may therefore offer a simple solution to the problem of inferring the role of roots in natural assemblages.

We found that there were no significant relationships between microbial data and soil functions, and this is another reason why structural traits are crucial in driving soil functions when the assemblage is newly established. When soils are shallow and nutrient poor, plants have a limited amount of resources to put into structural or metabolic tissues in chalk landscapes, and thus plant traits tend to fall into the more conservative end of the resource economics spectrum (Wright et al. 2004, de Vries et al. 2012). In chalk landscapes in particular, resources are limiting and species tend to remain quite small, with resources put into structural carbohydrates rather than more N rich, palatable leaves and roots. We found that taller plants in our study were associated with lower rates of N mineralization and nitrification, and consequently lower soil N availability. This runs counter to other literature that suggests that taller plants fall on the more exploitative, acquisitive end of the resource economics spectrum, so higher N mineralization rates would be expected (Laliberté and Tylianakis 2012, Grigulis et al. 2013). However, it is possible that the taller plants are poorly adapted to the hardships chalk imposes, particularly because they are likely to have higher requirements for nutrients and water, and that this may cause problems for acquisitive species, particularly early on in succession before plant–microbial relationships are established. Recent studies on chalk species showed that positive plant–soil feedback was associated with poor resistance to drought because the exploitative strategy of these plants meant severe problems and lack of structural integrity, which became problematic when drought occurred (Fry et al. 2018).

Vegetation composition of individual plots did not change significantly between planting in 2013 and 2015 when measures of soil function were taken. The trait groups that we present here do not fit neatly to the resource economics spectrum, in that higher SLA species, usually correlated with more acquisitive resource use, often had simpler tap rooted architectures, which we hypothesized would be more conducive to conservative strategies. Poorter and Remkes (1990) concluded that weak relationships between SLA and root characteristics in infertile conditions such as chalk could be explained by reduced competition for light, but increased belowground competition for nutrients, which decouples relationships between the above‐ and belowground organs. Support for this comes from other studies, where the relative importance of functional traits varies according to the availability of resources (Dyer et al. 2001). Furthermore, this departure from the plant economics spectrum may indicate that predicted relationships among traits may break down when the focus is on a limited geographic extent and/or specific ecosystem type (e.g., de la Riva et al. 2016). Indeed, the plants in this study were generally similar and the range of SLA values was relatively small.

A key finding of our study is that structural traits linked to plant height and rooting architecture are important in determining soil multifunctionality in the early stages of restoration. Plant assemblages with shallow complex root systems were associated with high values of multifunctionality in May, when plant growth was greatest, but in July shortly after peak biomass, tall plants were associated with lower rates of N cycling, and multifunctionality was consequently lower when the plant community was dominated by tall‐growing plants. There is a great deal of support for the usefulness of plant, or canopy height, for correlating with soil processes and management type (Pakeman 2011), but root architectural complexity could offer an equally important mechanistic link. We found that as root complexity increased, multifunctionality also increased. Fine roots are known to play an important role in nutrient cycling and resource capture (Jackson et al. 1997, Liu et al. 2017), and it is therefore possible that the finer root systems have more intimate contact with the soil environment and a high surface area for connecting microbes and root exudates, which are known to stimulate microbial activity and rates of nutrient and C cycling (Dijkstra et al. 2013). Further, such increases in nutrient availability may have enhanced plant nutrient supply, thereby contributing to a greater aboveground plant production and *R*_eco_ in this treatment (De Graaff et al. 2014, Meier et al. 2017). Rillig et al. (2015) suggest that high surface area increases root–fungal associations, increasing soil aggregation and stability. As roots become finer they become more acquisitive and N rich, and N cycling increases, in line with the proposed root economic spectrum (Roumet et al. 2016, Freschet et al. 2017). This is because a key function of fine roots is to access soil nutrients and water, rather than to stabilize the plant or to store C. Conversely, tap rooted systems, while deeper, have a higher volume to surface area ratio; as such, they have less contact with soil and microbes involved in nutrient cycling. This, in turn, enhances C sequestration through reducing accessibility of the organic matter to decomposition. Taken together, these results may indicate that soil multifunctionality could be assessed through the season using a simple screening procedure based on plant morphology.

A further important finding was that resistance of soil functions to drought was determined by rooting depth. Deeper roots delivered higher resistance when all soil functions were averaged, but for individual functions that were microbial driven and subject to rapid change, such as *R*_eco_ and N mineralization rate, a wide variety of rooting depths ensured higher resistance. Droughts in temperate regions tend to occur in a variety of small and larger rainfall inputs, with the smaller inputs rapidly evaporating from the surface. Shallow roots are likely to be poised to access these small inputs (Schwinning and Sala 2004). De Graaff et al. (2014) found that soil and root depth had a strong and consistent impact on C priming in switchgrass cultivars, which indicates that C cycling is more rapid and more C is lost to respiration when roots are in the surface soil layers. During long gaps between inputs, deeper roots are thought to be better able to not only reach water in deep soil layers, but also to employ “hydraulic lift” to redistribute water to neighbors in shallow soil (Vadez 2014). A key characteristic of lowland chalk grassland is that it has extremely shallow soil of high pH, which means that deep roots are largely plumbing cracks in the chalk substrate. The lack of viable soil below the surface could have important consequences for soil functions when drought occurs in these systems, though to our knowledge this has not been investigated.

The drought treatment had no effect on vegetation composition, but it did affect soil functions; concentrations of dissolved C and N in soil showed low resistance to the drought treatments, and recovery tended to coincide with a strong flush of microbial activity, which is consistent with the “Birch effect” that is commonly observed upon rewetting of soil (Birch 1958). Further, the increase in activity was long lasting; two months after the drought was alleviated, microbial‐driven processes such as decomposition, nitrification, and respiration were all higher than the control. It appears that the lack of recovery of these processes in the two months after the drought could be linked with the poor performance of tall plants in the latter half of the growing season. Additionally, we found few links between the plant community and the soil microbial community. We suggest that during early stages of restoration, soil functions are highly related to the composition of the plant community and their root traits, while plant‐microbial interactions form later on. In our study, there was no link between plant attributes and soil microbial community composition, suggesting that these links take many years to form (Morriën et al. 2017). In a well‐established chalk grassland system, Sayer et al. (2017) found that traits concerning plant tissue quality (leaf C to N ratio, specific leaf area and so on) had the strongest effect on microbial community compositional shifts when drought occurred. It is likely that both microbial community composition and plant traits related to tissue quality will become more important over successional time, in step with each other. Continuing our theory that the taller, more exploitative plants had been more adversely affected by drought because of their higher needs for water, and the lack of links with microbes at this stage of succession, it suggests that senescence and root sloughing from these large plants was adding labile, easily decomposed, plant material to the soil (Van Peer et al. 2004, Vogel et al. 2012). This material would be rapidly consumed by the “weedy” microbes that remained in the soil after the arable conversion. This notion is supported by the finding that decomposition rates in droughted plots were actually higher than in the control when FDvar plant perenniality was high, so life spans varied in the plots. This is because the majority of chalk species are perennial, so high divergence indicates the presence of annuals and biennials, both of which are likely to have higher tissue quality and a faster turnover through a more exploitative strategy (Fry et al. 2013).

## Conclusions

Here we demonstrate that plant functional traits based on structural features of both shoots and roots are effective predictors of soil functioning, multifunctionality, and resistance to perturbations in early successional grassland. Further, we demonstrate the importance of root traits, in that the sowing of functional trait groups characterized by fine‐rooted species consistently resulted in greater rates of soil C and N cycling. We considered a range of traits linked with plant structure and plant tissue quality, and the most consistent finding was that soil functions and multifunctionality are strongly dictated by plant structure and morphology in a restoring grassland. Microbial associations with plants occur later, and it is possible that the importance of tissue quality will increase, as microbial communities are highly dependent on inputs to soil of plant litter and root exudates, and this will have a cascading effect on soil functions. Our findings provide evidence that, at least during early stages of restoration, the sowing of plant communities based on their plant functional traits offers potential for enhancing restoration success, and that simple screening based on aboveground and belowground categorical traits can be useful in determining both reinstatement of soil functioning and resilience to perturbations.

## Data Availability

Data associated with this study are available through the NERC Environmental Information Data Centre at https://doi.org/10.5285/0e2bbef4-47db-43dc-849b-c7ce49d5bcec.

## Note

6 www.metoffice.gov.uk
