# Supplementary material for: Soil multifunctionality and drought resistance are determined by plant structural traits in restoring grassland
Source: Ecology. 2018 Aug 20;99(10):2260–71. doi: 10.1002/ecy.2437 (PMC6849565; doi:10.1002/ecy.2437)
Supplement: Supplementary file 1 [file ECY-99-2260-s001.pdf]

SUPPORTING INFORMATION. Soil multifunctionality and resistance of functions to drought are determined by plant structural traits in restoring grassland. Ellen L. Fry, Joanna Savage, Amy L. Hall, Simon Oakley, W. James Pritchard, Nicholas J. Ostle, Richard F. Pywell, James M. Bullock, and Richard D. Bardgett. *Ecology*. 2018.

APPENDIX S1

Detailed methods

Site attributes: Before the start of the experiment, the land (100 m x 100 m) was fenced to prevent livestock grazing, and treated with Roundup Pro Biactive 360 (active ingredient: Glyphosate 360 g l^{-1}) to kill vegetation. The exposed soil was a clay loam of approximately 9 cm depth, composed of a mineral substrate and calcareous (chalk) rubble (pH 8.11, organic C 42.0 kg ha^{-1}, N 2.3 kg ha^{-1}, phosphate (P) 15.5 mg l^{-1}). These C and N values are lower than average values for species-rich grassland on Salisbury Plain, (95.58 kg ha^{-1} ± 14.73, and 6.53 kg ha^{-1} ± 0.40 respectively, Fry *et al.* unpubl. data); as such, the soils of the site were depleted in both C and N, probably as a result of the continuous cultivation. The site was sheep-grazed for four weeks during December of each year of the experiment.

Creating the plots: To create plots that resembled the natural assembly of the plant community, we calculated the weight of seed to be added to each plot using the mean seed weight (Seed Information Database, Kew 2015), and the mean seedbank density (Leda-traitbase.org) of each species. Due to low availability of the different species, we applied a range of seeds and plug plants. Seed was acquired from Emorsgate Seeds (Norfolk, UK), and applied to the site in May 2013 by hand scattering. The plug plants were evenly divided between treatments and dug in randomly across each plot (Table S1).

Root ingrowth cores: These comprised two 3 mm mesh bags 4 cm in diameter that were buried 10 cm deep. They were filled with soil that had had all root material and coarse fragments removed (>2 mm). The ingrowth cores were inserted into the subplots in late May, then one was removed in mid-July the day after the shelters came down, and the other was removed in

September after eight weeks of recovery time. Roots were removed from the soil, washed thoroughly and dried, and root mass was calculated per 1 m^2 of soil.

Soil nutrients: Five small samples (5 cm diameter and no more than 9 cm deep) were collected per subplot using an augur down to the solid chalk layer, and bulked to create a composite sample, which was stored at 5 °C. These samples were weighed, then homogenised and passed through a 2 mm sieve, to remove gravel and roots. Gravel was weighed and the proportion calculated, and this was used to calculate bulk density ([mass dry soil-mass gravel]/[volume dry soil- volume gravel]; with gravel defined as largely calcite at a density of 2.71 mg m^{-3}; McKenzie *et al.* 2002). Soil pH was measured using a 1:1 v/v solution (Mettler Toledo FE20, Salford, UK). Gravimetric soil moisture was measured by calculating the percentage mass lost when 5 g fresh soil was incubated at 80 °C for 24 hours.

For soil sampled in September 2015, total C and N content was measured using soil dried at 80℃ for 24 hours, ground and then put through a total combustion analyser (Elementar vario EL, Hanau, Germany; Harrison & Bardgett 2004). At each sampling date, soil inorganic N availability was assessed as KCl extractable N (nitrate; NO_3-N and ammonium; NH_4-N), which involved using 25 ml 1 M KCl per 5 g soil and shaking for one hour at 150 rpm, before filtering through Whatman No 1 filter paper and analysing on a Seal AA3 autoanalyser at a 1:1 dilution (Allen 1989). Dissolved inorganic N (DIN) was determined using the same method, but substituting KCl for Milli-Q water. The water-extracted samples were also run on a Shimadzu TOC-L analyser to measure dissolved organic C (DOC). Soil extractable P was measured using Olsen's P method, before analysing using the autoanalyser as before (Olsen 1954). All values were multiplied by bulk density to gain accurate values of each nutrient in kg ha^{-1}. Potential rates of N mineralisation were ascertained by incubating 5 g of fresh soil at 25 °C for 14 days, before extracting with KCl and analysing as before. The potential rate of N mineralisation was calculated by subtracting N at day 0 from N at day 14, multiplying this value by bulk density, then dividing by 14 to get g N m^2 d^{-1}. Nitrification rate was ascertained using the same method, but only using NO_3-N (Bardgett *et al.* 2003).

Microbial biomass and stoichiometry: Microbial biomass C and N was determined using the chloroform-fumigation technique (Vance *et al.* 1987), using K_C and K_N factors of 0.35 and 0.45

respectively (Joergensen & Mueller 1996), whereas microbial community composition was measured using phospholipid fatty acid analysis (PLFA). Fatty acid methyl-esters were extracted from 1.5 g fresh soil using the method of Frostegård *et al.* (1991) and separated methyl-esters were analysed using an Agilent Technologies 7890B gas chromatograph with a DB5-MS column, which enabled identification and quantification of individual bacterial and fungal fatty acid methyl-esters by comparison with a standard methyl ester mix which ranged from C11 to C20 (Sigma-Aldrich, UK). Individual fatty acid methyl-esters were expressed as µg PLFA g^{-1} dry soil. We used 14:0, i15:0, a15:0, i16:0, 16:1ω9, 16:0, i17:0, a17:0, cy17:0, 17:0, 18:1 ω9, 18:1 ω7, 18:0 and cy19:0 as bacterial biomarkers (Federle, 1986; Frostegård *et al.*, 1993) and 18:2 ω6 as a fungal biomarker (Federle, 1986), and the ratio of total fungal to bacterial biomass was calculated (Bardgett *et al.* 1996).

Decomposition: Litter bags were made using 1 mm aperture vinyl mesh (10 cm x 10 cm). Senescent plant material used was taken from a mixed natural grassland sward (*Bromus erectus* sward with typical subcommunity; CG3a classification; Rodwell 1992) adjacent to the study site and approximately 2 g of litter was added to each bag. Upon collection, soil and external litter was brushed off, and fresh growth from the subplots was removed. The remaining litter was then dried at 80 °C for 24 hours and weighed. Decomposition rate was measured as percentage mass loss per day.

Table S1: Species allocation by functional groupings. Species derived from CG3a classification. Species in red were planted as seed in the plots, species in blue were added as plug plants. Black means that the species were unobtainable.

FG1	FG2	FG3
Achillea millefolium	*Acinos arvensis*	*Anthyllis vulneraria*
Agrimonia eupatoria	*Agrostis stolonifera*	*Avenula pratensis*
Arctium minus	*Agrostis capillaris*	*Avenula pubescens*
Arrhenatherum elatius	*Anthoxanthum odoratum*	*Bromopsis erecta*
Asperula cynanchica	*Bellis perennis*	*Campanula glomerata*
Blackstonia perfoliata	*Briza media*	*Centaurea nigra*
Carduus nutans	*Campanula rotundifolia*	*Cynosurus cristatus*
Carlina vulgaris	*Carex caryophyllea*	*Danthonia decumbens*
Centaurea scabiosa	*Carex flacca*	*Festuca pratensis*
Centaurium erythraea	*Cerastium fontanum*	*Festuca ovina*
Crepis capillaris	*Cirsium acaule*	*Galium mollugo*
Dactylis glomerata	*Clinopodium vulgare*	*Gentianella amarella*
Daucus carota	*Cruciata laevipes*	*Hypochaeris maculata*
Euphrasia officinalis agg.	*Festuca rubra agg.*	*Knautia arvensis*
Galium verum	*Filipendula vulgaris*	*Koeleria macrantha*
Helianthemum nummularium	*Hieracium pilosella (planted P. aurantiaca)*	*Leucanthemum vulgare*
Holcus lanatus	*Hippocrepis comosa*	*Lolium perenne*
Hypochaeris radicata	*Lathyrus pratensis*	*Onobrychis viciifolia*
Leontodon hispidus	*Plantago media*	*Picris hieracioides*
Linaria vulgaris	*Poa subcaerulea*	*Polygala vulgaris*
Linum catharticum	*Potentilla reptans*	*Pulsatilla vulgaris*
Lotus corniculatus	*Primula veris*	*Ranunculus acris*
Medicago lupulina	*Prunella vulgaris*	*Ranunculus bulbosus*
Ononis repens	*Ranunculus repens*	*Reseda lutea*
Ononis spinosa	*Taraxacum officinale*	*Rumex acetosa*
Origanum vulgare	*Thymus pulegioides*	*Stachys officinalis*
Phleum bertolonii	*Trifolium repens*	*Succisa pratensis*
Phleum pratense	*Trifolium pratense*	*Thesium humifusum*
Pimpinella saxifraga	*Viola hirta*	*Thymus praecox*
Plantago lanceolata		*Tragopogon pratensis*
Poa pratensis sens lat		*Trisetum flavescens*
Sanguisorba minor		*Veronica chamaedrys*
Scabiosa columbaria		*Vicia cracca*
Serratula tinctoria		

Figure S1: Average trait mean for each functional group based on hierarchical cluster analysis. a) Plant height, ($F_{2,80}=6.30$, p=0.003), b) Perenniality, ($F_{2,80}=7.25$, p=0.001), c) Root architecture class; 1: fibrous root, 2: adventitious, 3: bulbous, 4: nodal, 5: rhizomatous, 6: stoloniferous, 7: tap rooted ($F_{2,80}=47.03$, p<0.001), d) Specific leaf area ($F_{2,80}=7.50$, p=0.001), e) Root depth ($F_{2,80}=47.22$, p<0.001), f) Mycorrhizal association, no significant difference.

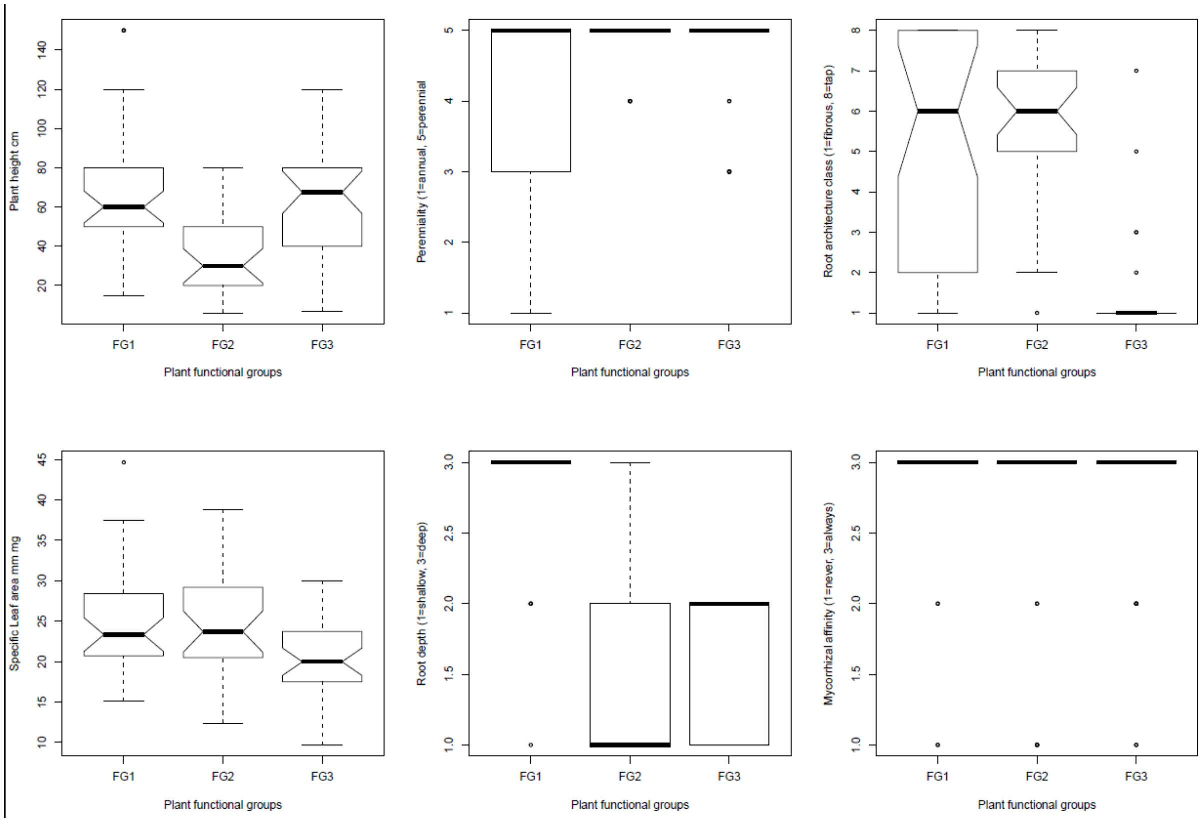

Figure S2: Realised species distribution into functional groups in July 2013 compared with plot identity. For example, the FG1 column should be 100% FG1 species, but some invasion has taken place.

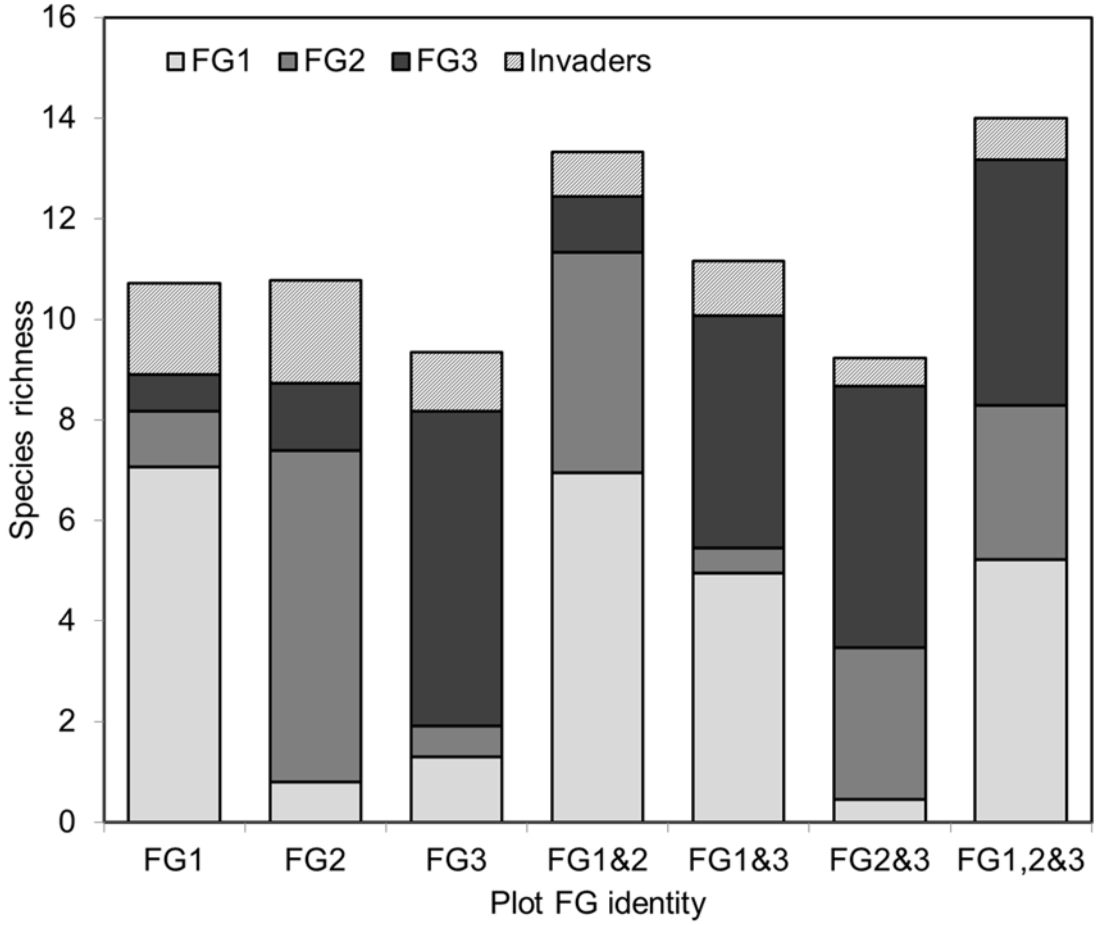

Figure S3: Correlation matrix of a) realised traits (community weighted means), and b) functional divergence of traits (FDvar) in July 2015.

a)

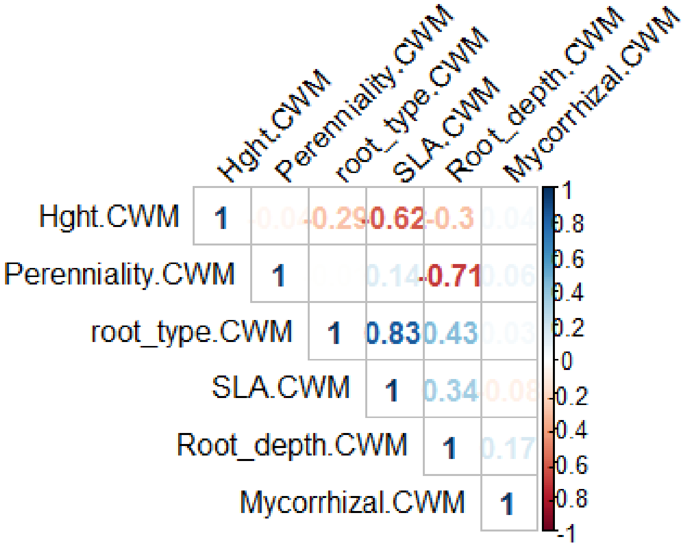

b)

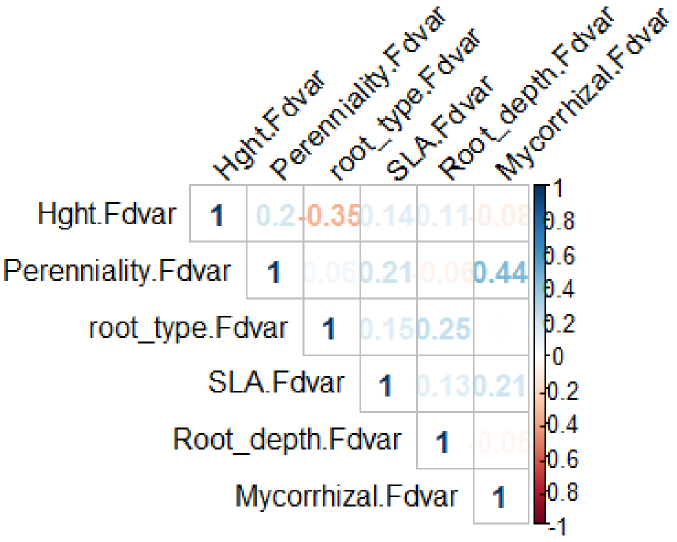

Figure S4: Above- and belowground biomass for each functional group combination in September 2015 (AGB and BGB respectively).

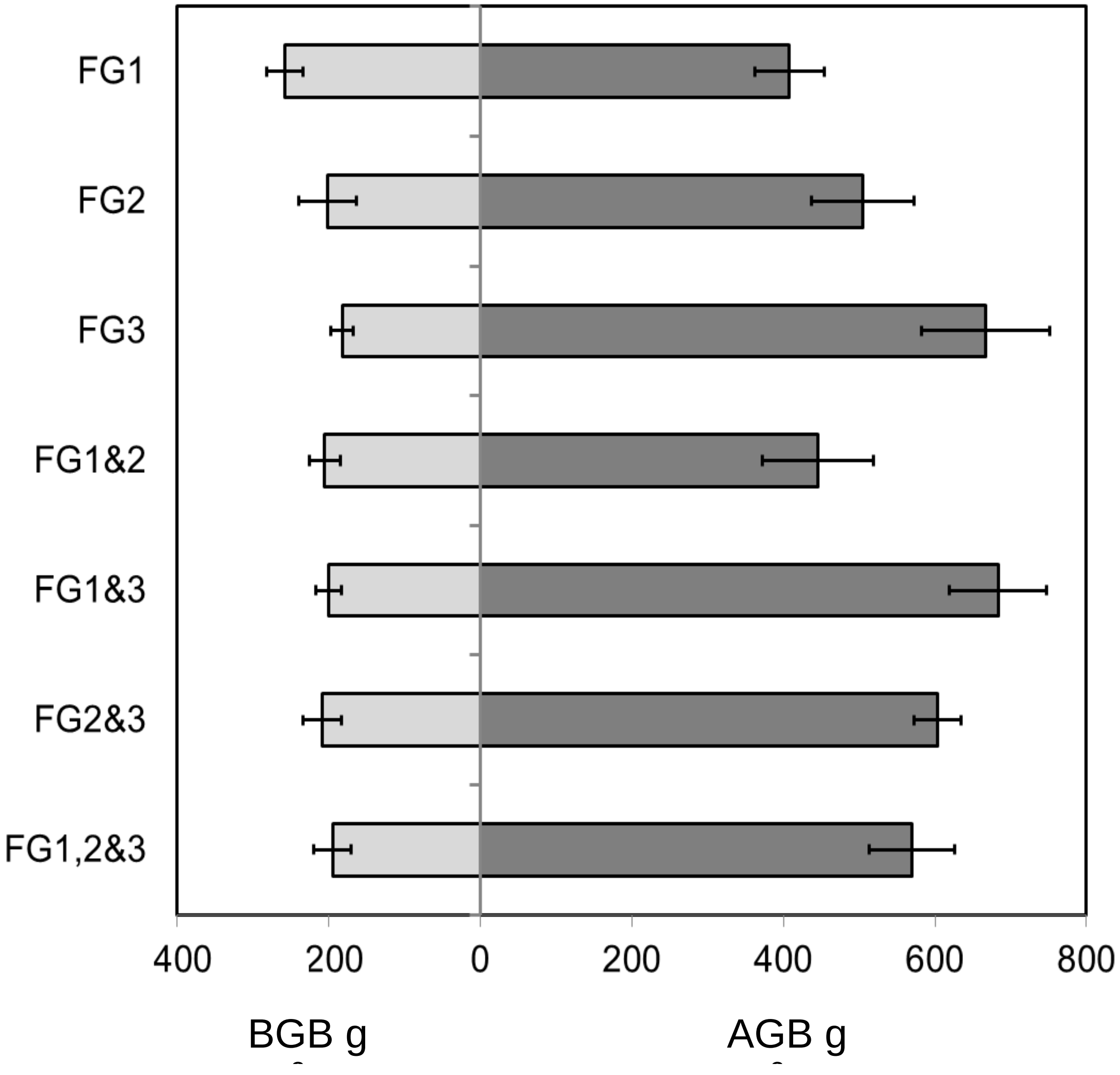

Table S2: Importance of Random Forest mean predictors of individual ecosystem functions for July 2015. Values for each variable denote % increase in mean square error (MSE) relative to a randomised dataset, and negative numbers indicate that the randomised variable performed better than the real data. Bold type refers to significance at the $p<0.05$ level. Goodness of fit (R^2) and cross-validation of the model (MSE) are presented.

a) May (before roofs)

	Model MSE	Model R^2	Soil Moisture Content	CWM Plant Height	CWM Perenniality	CWM Root architecture	CWM Root depth	CWM SLA	CWM Myco affinity	FDvar Plant Height	FDvar Perenniality	FDvar Root architecture	FDvar SLA
NO_3-N (H_2O)	0.072	0.833	4.31	10.04	1.24	7.23	1.48	**11.05**	**15.18**	10.23	-0.65	**12.94**	**5.71**
NH_4-N (KCl)	0.034	0.802	5.03	6.19	7.55	5.01	2.32	0.89	4.17	9.67	**8.94**	0.65	-1.38
Mineralisation rate	0.066	0.799	-4.15	2.46	**6.33**	3.62	-1.51	13.25	14.35	6.21	1.15	**3.86**	9.45
Nitrification rate	0.086	0.797	-2.73	4.53	**5.90**	4.09	-3.41	13.41	15.90	9.48	1.51	**5.03**	10.53
PO_4-P	0.203	0.808	12.49	7.13	3.98	10.19	1.58	2.36	10.62	14.86	3.74	**6.92**	11.24
Respiration	0.160	0.827	-0.03	4.47	1.01	1.97	3.77	1.94	**21.69**	13.61	4.21	4.20	8.12
Photosynthetic rate	0.294	0.804	2.21	3.83	5.82	7.56	**2.11**	**6.82**	5.08	1.39	5.14	**12.64**	-1.96
NEE	0.310	0.721	0.98	5.91	5.51	8.28	**4.76**	6.11	3.71	0.50	6.15	3.73	3.18
Microbial biomass	0.153	0.813	**12.85**	**14.97**	4.53	0.22	5.47	6.74	3.21	6.94	4.37	2.45	1.52
Bacteria	0.204	0.828	**25.43**	7.79	5.96	1.01	6.86	7.77	2.20	7.79	3.46	-1.44	1.92
Fungus	0.087	0.805	**11.80**	3.93	11.18	2.47	-2.85	0.45	-0.30	7.08	-0.14	-0.23	-1.28

b) July (after roofs removed)

	Model MSE	Model R^2	SMC	Roof	CWM Plant Height	CWM Perenniality	CWM Root architecture	CWM Root depth	CWM SLA	CWM Myco affinity	FDvar Plant Height	FDvar Perenniality	FDvar Root architecture	FDvar SLA
DOC	0.094	0.765	0.14	**1.32**	3.04	7.74	3.29	1.07	1.18	1.71	-1.02	0.35	**23.20**	2.15
Microbial biomass C	0.051	0.814	3.96	-4.31	-3.52	-0.91	10.61	11.07	3.23	4.16	2.31	-0.56	**11.77**	1.19
Decomposition rate	0.073	0.771	-3.15	**11.09**	**6.89**	4.21	1.57	1.27	4.88	4.46	1.19	8.13	-5.30	0.21
Total N	0.042	0.796	-2.91	-3.52	10.35	3.38	6.78	0.20	-3.81	4.94	4.83	10.28	2.86	-0.50
NO_3-N (H_2O)	0.103	0.836	**17.25**	**-1.03**	**5.49**	2.49	-1.36	7.04	**19.70**	6.60	0.42	-2.07	-1.29	10.67
NH_4-N (KCl)	0.124	0.815	-1.33	-1.47	**8.67**	0.63	**3.39**	-4.08	3.23	13.90	12.03	2.17	-1.67	3.75
Mineralisation rate	0.164	0.801	-4.76	-1.52	**15.98**	4.41	12.94	-2.05	5.19	8.20	9.63	7.46	1.34	5.03
Nitrification rate	0.190	0.801	-4.78	1.38	**12.96**	5.32	6.81	1.04	2.27	11.49	13.42	2.86	0.18	7.41
PO_4-P	0.203	0.778	-3.03	0.29	6.21	**2.97**	-2.43	5.15	2.87	4.44	5.76	-4.04	0.52	2.55
Respiration	0.184	0.830	1.89	-1.30	**6.81**	**6.95**	5.72	5.91	0.94	**14.03**	6.81	20.81	9.26	3.35
Photosynthetic rate	0.135	0.810	-1.54	-0.45	8.16	10.97	2.66	1.59	**5.97**	8.60	**11.34**	13.18	-1.45	3.37
NEE	0.212	0.823	-2.67	0.91	12.71	10.66	7.34	4.65	**13.53**	5.54	6.78	6.86	-4.44	1.70
Microbial biomass	0.218	0.802	-2.17	-3.07	7.79	10.63	4.14	5.60	2.36	4.29	6.47	1.97	8.61	7.00
Bacteria	0.167	0.805	-3.19	0.91	8.88	12.10	7.24	1.88	2.60	0.52	6.57	2.18	13.26	-2.15
Fungus	0.041	0.795	2.94	-2.63	1.11	6.79	-3.49	8.10	-0.12	5.17	3.55	5.97	6.87	3.88

c) September (eight weeks after roofs removed)

	Model MSE	Model R^2	SMC	Roof	CWM Plant Height	CWM Perenniality	CWM Root architecture	CWM Root depth	CWM SLA	CWM Myco affinity	FDvar Plant Height	FDvar Perenniality	FDvar Root architecture	FDvar SLA
DOC	0.166	0.817	-2.36	1.03	**10.07**	3.73	13.00	2.30	10.00	6.82	-1.79	1.44	-0.01	-6.01
Microbial biomass C	0.207	0.809	**12.23**	-3.85	0.75	7.49	4.03	4.80	1.92	-1.99	-2.27	-2.69	4.81	-8.09
Decomposition rate	0.051	0.803	-4.26	-1.33	-0.45	-1.18	4.97	0.21	-5.28	4.11	**-0.94**	-8.97	6.29	-2.12
Total N	0.184	0.809	-3.19	-0.84	**10.43**	0.95	**4.85**	1.73	**-0.46**	6.77	1.83	-0.33	-1.56	-2.18
NO_3-N (H_2O)	0.169	0.826	0.37	4.18	**18.63**	2.21	-0.98	2.54	-1.67	5.67	2.62	-0.84	6.74	-4.11
NH_4-N (KCl)	0.041	0.820	**11.12**	-1.83	7.25	6.70	5.53	-4.87	-2.99	7.41	2.76	0.01	4.04	5.12
Mineralisation rate	0.238	0.811	1.60	0.49	**12.67**	2.59	-1.24	**5.43**	-1.25	-0.14	11.11	-1.76	3.41	2.77
Nitrification rate	0.251	0.809	2.95	1.89	**12.46**	6.37	-0.06	5.67	-1.10	2.10	12.71	0.07	5.11	1.43
PO_4-P	0.154	0.819	-4.20	**10.27**	**1.70**	-3.24	7.65	**15.86**	-2.23	2.95	0.45	-0.04	-3.98	3.32
Respiration	0.078	0.834	6.07	1.03	6.51	1.30	7.52	7.39	11.60	6.85	-0.29	19.43	1.98	1.13
Photosynthetic rate	0.043	0.815	**13.12**	-4.43	7.22	-1.40	3.56	4.70	-1.66	6.49	-1.74	-3.40	11.50	2.81
NEE	0.061	0.829	**14.30**	-1.95	5.64	-2.84	0.11	**11.30**	3.75	4.15	-0.42	-0.47	16.37	2.91
Microbial biomass	0.164	0.804	0.46	-2.37	5.32	6.53	4.39	0.48	-1.39	4.95	-1.62	10.83	-1.21	-1.49
Bacteria	0.146	0.815	7.67	-2.91	9.56	8.17	2.36	3.58	-1.14	**10.21**	-2.24	4.78	-3.09	-1.31
Fungus	0.478	0.759	2.93	-1.63	4.18	4.67	-2.87	4.20	4.39	3.56	-2.20	1.85	-2.79	4.12

REFERENCES FOR APPENDIX S1

Allen, S. E. 1989. Chemical analysis of ecological materials. Blackwell Scientific Publications, Oxford, United Kingdom.

Bardgett, R. D., P. J. Hobbs, and A. Frostegård. 1996. Changes in soil fungal:bacterial biomass ratios following reductions in the intensity of management of an upland grassland. Biology and Fertility of Soils 22:261–264.

Bardgett, R. D., T. C. Streeter, and R. Bol. 2003. Soil microbes compete effectively with plants for organic-nitrogen inputs to temperate grasslands. Ecology 84:1277–1287.

Box, G. E. P. and D. R. Cox. 1964. An analysis of transformations (with discussion). Journal of the Royal Statistical Society B 26:211–252.

Díaz, S., S. Lavorel, F. De Bello, F. Quétier, K. Grigulis, and T. M. Robson. 2007. Incorporating plant functional diversity effects in ecosystem service assessments. Proceedings of the National Academy of Sciences of the United States of America 104:20684–20689.

Federle, T. W. 1986. Microbial distribution in soil - new techniques. *in* Perspectives in Microbial Ecology. F. Megusar, and M. Gantar, *editors*. Proceeding of the Fourth International Symposium on Microbial Ecology. Slovene Society for Microbiology, Ljubljana.

Frostegård, Å., A. Tunlio, and E. Bååth. 1991. Microbial biomass measured as total lipid phosphate in soils of different organic content. Journal of Microbiological Methods 14:151–163.

Frostegård, Å., E. Bååth, and A. Tunlio. 1993. Shifts in the structure of soil microbial communities in limed forests as revealed by phospholipid fatty acid analysis. Soil Biology and Biochemistry 25:723–730.

Harrison, K. A. and R. D. Bardgett. 2004. Browsing by red deer negatively impacts on soil nitrogen availability in regenerating native forest. Soil Biology and Biochemistry 36:115–126.

Joergensen, R. G. and T. Mueller. 1996. The fumigation-extraction method to estimate soil microbial biomass: calibration of the KEN value. Soil Biology and Biochemistry 28:33–37.

Mason, N. W. H., K. MacGillivray, J. B. Steel, and J. B. Wilson. 2003. An index of functional diversity. Journal of Vegetation Science, 14: 571–578. doi:10.1111/j.1654-1103.2003.tb02184.x

McKenzie, N., K. Coughlan, and H. Cresswell. 2002. Soil physical measurement and interpretation for land evaluation. Australian Soil and Land Survey Handbooks Series 5. CSIRO Publishing, Victoria, Australia.

Nakagawa, S. and H. Schielzeth. 2013. A general and simple method for obtaining R2 from generalized linear mixed-effects models. Methods in Ecology & Evolution 4:133–142.

Olsen, S. R., C. V. Cole, F. S. Watanabe, and L. A. Dean. 1954. Estimation of available phosphorus in soils by extraction with sodium bicarbonate. USDA Circular 939. Washington, DC: Government Printing Office.

Vance, E. D., P. C. Brookes, and D. S. Jenkinson. 1987. An extraction method for measuring soil microbial biomass C. Soil Biology and Biochemistry 19: 703–707.

Venables, W. N. and B. D. Ripley. 2002. Modern Applied Statistics with S. Fourth Edition. Springer, New York, New York, USA. ISBN 0-387-95457-0

Yee, T. W. 2010. The VGAM package for categorical data analysis. Journal of Statistical Software 32:1–34.
